# Seroprevalence of transfusion transmitted infection among blood donors at Jijiga blood bank, Eastern Ethiopia: retrospective 4 years study

**DOI:** 10.1186/s13104-016-1925-6

**Published:** 2016-02-27

**Authors:** Yusuf Mohammed, Alemayehu Bekele

**Affiliations:** Public Health Emergency Management, Ethiopian Somali Regional Health Bureau, P.O. Box 238, Jijiga, Ethiopia; Ethiopian Public Health Association, P.O. Box 7176, Addis Ababa, Ethiopia

**Keywords:** Jijiga blood bank, Transfusion transmission infection, Hepatitis B, Ethiopian Somali region

## Abstract

**Background:**

A transfusion transmissible infection (TTI) is any infection that is transmissible from person to- person through parenteral administration of blood or blood products. The magnitude of transfusion-transmitted infections (TTI) varies from country to country depending on TTI’s load in that particular population. Measuring their severity, WHO (World Health Organization) has recommended pre-transfusion blood test for Human immunodeficiency virus (HIV), Hepatitis B virus (HBV), Hepatitis C Virus (HCV) and Syphilis as mandatory. The aim of the current study was to assess the trend and prevalence of TTI among blood donors in Jijiga Blood Bank between 2010 and 2013.

**Methods:**

A Retrospective cross-sectional study was conducted by reviewing the records from 2010 to 2013 at Jijiga Blood Bank. All blood donors who presented to the blood bank and screened for TTI during the study period were included. The data was collected, entered and analyzed using Epi Info 3.5.1 & Microsoft Excel 2007. The descriptive statistics were determined in means of percentages. Chi-square was used for trend analysis and p-value was used to declare the statistical significance between the variable.

**Result:**

There were a total of 4224 people donated blood during study period. Males formed the majority of the donor population accounting for 4171 (98.7 %). Majority 4139 (98 %) of donors were Replacement donors. The overall prevalence of transfusion-transmitted infection was 487/4224 (11.5 %). The prevalence for HBsAg, HCV, HIV, & Syphilis antibodies was 460 (10. 9 %), 17 (0.4 %), 6 (0.1 %) and 4 (0.1 %) respectively. Majority 460/487 (94.5 %) of infection was HBsAg. Statistically significant difference was observed in number of donation as well as sero-positivity from year 2010 to 2013 (Chi-square 9.24, p value = 0.02), in Trends of HBsAg from year to year (Chi-square 11.14, p value = 0.01), HIV virus was seen as the age of donors increases (Chi-square 8.37, p value = 0.01) and There was also statistically significance difference (p value = 0.01) in prevalence of HBsAg distribution by sex.

**Conclusion:**

The present study clearly documents high Seroprevalence (487 out of 4,224, 11.5 %) of TTI, low percentage of voluntary donors and low participation of female donors. Promoting the culture of voluntary donors, recruitment of female blood donors and proper testing of donor’s blood by using standard methods are recommended.

## Background

Blood transfusion is a life-saving intervention and millions of lives are saved each year globally through this procedure. However, blood transfusions are associated with certain risks which can lead to adverse consequences. It may cause acute or delayed complications and carries the risk of the trans- mission of infections. Globally, more than 81 million units of blood are donated each year [1].

Blood transfusion is a therapeutic procedure, as there is no genuine substitution. But contaminated blood transfusion can transmit infectious diseases and can be fatal instead of saving life [2]. Evaluation of data on the prevalence of transfusion transmissible infections (TTIs) namely HIV, HBV, HCV and syphilis antibodies among blood and plasma donors permit an assessment of the occurrence of infections in the blood donor population and consequently the safety of the collected donations. It also gives an idea of the epidemiology of these diseases in the community. Transfusion associated infections continue to be a big threat [3].

Transfusion transmissible infections can be classified as viral, bacterial and parasitic infections. The most commonly encountered transfusion infection is of viral origin. In many cases, post transfusion diseases have been caused by human immunodeficiency virus (HIV), hepatitis B and C virus [4]. Prevalence of HBV infection varies greatly in different parts of the world. The World Health Organization (WHO) has classified HBV prevalence into high endemicity (>8 %), intermediate (2–7 %) and low endemicity (<2 %) [5].

Worldwide about 350 million people have chronic hepatitis B virus (HBV) infection, and about 125 million have been infected with hepatitis C virus (HCV), putting viral HBV and HCV infection among the world’s greatest infectious disease problems. These diseases are therefore important candidates for public health measures aimed at prevention, early diagnosis and treatment [6].

The discovery of (TTIs) has heralded a new era in blood transfusion practice worldwide with emphasis on two fundamental objectives, safety and protection of human life. Blood safety remains an issue of major concern in transfusion medicine in Ethiopia [7].

Estimating the prevalence of TTIs, namely HBV, HCV, HIV and syphilis antibodies or antigen, among blood donors can reveal the problem of unnoticeable infections in healthy-looking members of the general population and also provide data that is important in formulating the strategies for improving the management of a safe blood supply. In addition it can give us a guide to the magnitude of some sexually transmitted infections in the community [1, 8, 9].

In Jijiga, the main source of blood donation is replacement donors and most of them are patient’s relatives or friends. Proper screening of blood and selection of the donors is very important to insure a safe blood supply. It is mandatory to test each donor’s blood for syphilis by a Venereal Disease Reference Laboratory (VDRL), and for HBsAg, anti-HCV, and anti-HIV.

Available data on the seroprevalence and distribution of these blood borne pathogens is not available or no study has been done. The purpose of this study was to estimate the prevalence of serological markers of HBV, HCV, HIV, and syphilis antibodies among blood donors in Jijiga blood bank in Jijiga.

## Methods

### Study area

The data was collected from the blood bank of Jijiga. Jijiga blood bank serves Karamara hospital (Hospital serving as Referral Hospital in Ethiopia Somali Region). The blood bank is situated in the Jijiga town, and is found 626 km east from the capital city, Addis Ababa. Karamara hospital Provides health services to the local as well as for population coming from different zone of the region.

### Study design

It was blood bank based retrospective analysis of consecutive blood donors’ summary monthly records from January 2010 to December 2013 (4 years) conducted at the regional blood transfusion center (Jijiga blood bank).

### Study population

Blood donors attended the Jijiga blood bank during 4 years period and those screened for hepatitis B, hepatitis C, HIV and syphilis antibodies.

### Study variable

The dependent variable of this study was HIV-, HBsAg, HCV, and syphilis test result, whereas the independent variables were sex, age.

### Laboratory tests

During 4 years blood sample was collected and tested in the Jijiga blood bank of Ethiopian Somali Region. HBsAg, anti-HCV, was tested by Microwell ELISA Test J. Mitra and Co Pvt Ltd. India the kit has a sensitivity of 100 % and specificity of 99.7 %. Anti-HIV were screened by using ELISA (Vironstiks) kit developed by bioMereux SA and Company Ltd., Marcy-IEtoile, France) has a sensitivity of 100 % and specificity of 99.7 %. Laboratory diagnosis for syphilis: serum from all donors was tested for the presence of treponemal antibodies using rapid plasma reagin test (RPR) following the manufacturer’s instructions (RPR, Wampole Laboratories, Princeton, NJ, USA).

### Statistical analysis

The data was collected, entered and analyzed using Epi Info 3.5.1 and Microsoft Excel 2007. The data were double entered; validated for data entry errors and subsequently analyzed. We presented our data in the form of tables. The descriptive statistics were determined in means of percentages. Chi square was used for trend analysis and *P* value was used to calculate statistical significances.

### Ethical issues

The study was approved by our institutional ethical committee and Curative Core process of Regional Health Bureau. However, because of the nature of the study (retrospective review of blood donors’ records), informed consent was not got from the study subjects.

### Limitation

The current study was based on retrospective review of monthly summary record at Jijiga blood bank, which limits the independent variables to only sex, age. Low female participation and generalization to the general blood donors and the absence of confirmatory tests for HIV, HBV and HCV are also among limitation.

## Results

### Socio demographic characteristics

Starting from January 2010 to December 2013, the total numbers of people gave blood were 4224. Among the donors visited Jijiga blood bank over past 4 years, male constitutes the majority 4171 (98.7 %) of the donors, while females make up 53 (1.3 %). The most common age group of donors was found to be 26–35 years (46 %) followed by age group of 16–25 years (27.9 %), while the least age group was >55 (0.4). Majority 4139 (98 %) of donors were replacement donors, while voluntary donors constitutes 2 % (Table 1).

**Table 1:** Socio-demographic characteristics of blood donors at Jijiga blood bank, Eastern Ethiopia from January 2010 to December 2013 (n = 4224)

Age group	Number of donated	Percentage (%)
16–25	1208	28.6
26–35	1965	46.5
36–45	836	19.8
46–55	197	4.7
>55	18	0.4
Total	4224	100.0
Sex		
Male	4171	98.7
Female	53	1.3
Total	4224	100.0
Types of donor		
Replacement	4139	98.0
Voluntary	85	2.0
	4224	100.0

### Trends of transfusion transmissible infection

Out of 4224 blood units collected, 487 units that tested positive for any of the TTI tested giving an overall positivity rate 11.5 %. No co-infection reported during this study period. Of all the TTI, hepatitis B form majority of infection 460/4224 (10.9 %), followed by hepatitis C 17 (0.4 %), while the least percentage was HIV and syphilis 6 (0.1 %), 4 (0.1 %) respectively. High percentage of TTI was reported in 2010 (14.1 %), followed by 2012 (12.4 %), while least was reported on 2011 (10.1 %). High percentage (13.9 %) of HBV was reported in 2010, followed by 2012 (11.6 %), the least was reported in 2011 (9.4 %). There was statistically significant (Chi square = 9.24 P value = 0.02) change in sero-positivity from year 2010 to 2013. Trends of Hepatitis B also statistically significant from year to year (Chi square = 11.14 P value = 0.01). All TTIs types were reported in 2011 and 2013, while syphilis cases were not reported in 2010 and 2012 (Table 2). When grouping (HCV, HIV, and syphilis) by years, the percentage was increasing from year 2010 (0.3 %), 2011 (0.6 %), 2011 (0.8 %), and decline in 2013 (0.7 %), but there was no statistically significant change (P value = 0.576) (Table 2).

**Table 2:** Year wise infected cases of HIV, HBV, HCV and syphilis from January 2010 to December 2013 at Jijiga blood bank, Eastern Ethiopia (n = 4224)

Year	Total tested	HBsAg + ve	HCV + ve	HIV + ve	Syphilis +ve	(HCV, HIV, Syphilies +ve)	Total +ve
2010	729	101 (13.9 %)	1 (0.1 %)	1 (0.1 %)	0 (0)	2 (0.3 %)	103 (14.1 %)
2011	774	73 (9.4 %)	3 (0.4 %)	1 (0.1 %)	1 (0.1 %)	5 (0.6 %)	78 (10.1 %)
2012	1136	132 (11.6 %)	8 (0.7 %)	1 (0.1 %)	0 (0)	9 (0.8)	141 (12.4 %)
2013	1585	154 (9.7 %)	5 (0.3 %)	3 (0.2 %)	3 (0.2 %)	11 (0.7 %)	165 (10.4 %)
		P = 0.01	P = 0.24	P = 0.92	P = 0.33	P = 0.567	P = 0.02

### Seroprevalence of HIV, HBV, HCV and syphilis

Highest prevalence of transfusion transmitted infection was within age group 36–45 years (12.8 %) followed by >45 (12.1 %), and the least affected age group were 16–35 (11.2 %). HBsAg and HCV was reported from all age group of donors, while HIV and syphilis was not seen in all age group >45. There was no statistically significant change in TTI as the age of donors increased except for HIV (P value = 0.01).

All types of TTI were reported from male donors, while HBsAg and syphilis was not reported from female’s donors. From all types of TTI only HBV (P value = 0.01) show statistically significant difference between male and females donors (Table 3).

**Table 3:** Distribution of transfusion transmitted infection by socio demographic factors at Jijiga blood bank, Ethiopian Somali Region between 2010 and 2013 (n = 4224)

Socio demographic variable	Tested	HBsAg +ve (%)	HCV +ve (%)	HIV +ve (%)	Syphilis +ve (%)	Total
Age group						
16–35	3173	338 (10.7 %)	11 (0.3 %)	2 (0.1 %)	3 (0.1 %)	354 (11.2 %)
36–45	836	97 (11.6 %)	5 (0.6 %)	4 (0.5 %)	1 (0.1 %)	107 (12.8 %)
>45	215	25 (11.6 %)	1 (0.5 %)	0 (0)	0 (0)	26 (12.1 %)
	4224	460 (10.9 %)	17 (0.4 %)	6 (0.1 %)	4 (0.1 %)	487 (11.5 %)
Statistical significance		P = 0.68	P = 0.58	P = 0.01	P = 0.87	P = 0.4
Sex						
Male	4171	460 (11 %)	16 (0.4 %)	5 (0.1 %)	4 (0.1 %)	485 (11.6 %)
Female	53	0 (0)	1 (1.9 %)	1 (1.9 %)	0 (0)	2 (3.8 %)
Total	4224	460 (10.9 %)	17 (0.4 %)	6 (0.4 %)	4 (0.1 %)	487 (11.5 %)
Statistical significance		P = 0.01	P = 0.08	P = 0.07	P = 0.95	P = 0.07

During our study, majority 460/487 (94.5 %) of infection was HBsAg, followed by HCV 17 (3.5 %), HIV 6 (1.2 %), the least was syphilis 4 (0.8 %) (Table 4).

**Table 4:** Distribution of transfusion transmitted infection by frequency at Jijiga blood bank, Ethiopian Somali Region between 2010 and 2013 (n = 4224)

Types TTTI	Number	Percentage (%)
HBV	460	94.5
HCV	17	3.5
HIV	6	1.2
Syphilis	4	0.8
Total	487	100

## Discussion

The results of this study showed the prevalence of major TTIs (HBV, HCV, HIV and syphilis antibodies) among blood units donated in Jijiga blood Bank of Ethiopian Somali Region from 2010 to 2013. In this study over the 4 year period, the total annual number of blood donations tested had increased steadily with a cumulative total of 4224.

In our study majority of the donors (98 %) were males which is similar the study done by Tessema et al. [7], by Baye et al. 96.3 % [10] Nwankwo et al. 98 % [11] Koram et al. 97.41 % [12], and Ismail et al. Libya 99.6 % [13].

The majority of age group donating blood in our study was those ranging from 26 to 35 years (46 %). This differs from the figures published by the World Health Organization (WHO) which reported that 45 % of donors were aged 25 or less [14]. Our study finding showed us that a lot of awareness creation activity targeting younger age group is needed.

In this study the overall prevalence of TTI among blood donors in Jijiga blood bank over past 4 years was 487 (11.5 %), This was higher, the findings from neighboring Eritrea 3.8 % [2], study done by Manzoor et al. and was 9.9 % [1], TTI in Brunei Darussalam et al. 1.49 % [14], Yemen by Saghir et al. 2.35 % [8], among blood donors in Kassala, eastern Sudan by Abdallah and Ali 3 % [15], Tessema et al. 9.5 % [7] by Baye et al. 6.2 % [10]. The higher prevalence in our case may be due to the difference in the health care system in different study setting.

Our finding was lower when compared to A study in Teaching Hospital of Islamabad by Waheed et al. was 14.34 % [16], northwest Ethiopia 43.2 % [17], Nwankwo et al. in Nigeria (19.3 %) [11]. The reason for the relatively lower rate of seroprevalence of TTI in this study compared with other studies may be because of existence of different magnitude of risk factor for contracting transfusion transmitted infection and may be also the screening technique used.

Hepatitis B is one of the most infectious diseases; it has infected around 2 billion people worldwide, including an estimated 400 million chronically infected cases. It is also hyper endemic in sub-Saharan Africa and Asia [2]. In our study the prevalence rate of HBs Ag was 10.9 %. This figure is comparable with Nwankwo et al. 10.9 % [11], 10.9 % among street dwellers in Gondar city [18]. The current study finding classify study area as WHO high endemic classification [19].

Our study finding is higher than 3.5 % by Lavanya et al. [20] in Pakistan and 2.58 % neighboring Eritrea 4 %, Tessema et al. Gondar University Teaching Hospital 4.7 % [7], Baye et al. 2007 6.2 % [10], Sharew et al. 4.66 % [21] Anagaw et al. 6 % [22] Negero et al. 5.7 % [23] among VCT clients, Shiferaw et al. 6.3 % [24] among medical waste handlers, 4.9 % [25] among pregnant women attending ANC and also higher than 7 % [26] community based survey in Addis Ababa by Abebe and others.

However our current finding is lower when compared to study done on donors at Felege Hiwot referral hospital 25 % [17] Buseri et al. Nigeria 18.6 % [27], Taye et al. 22.3 % [28], Bereka Medical center, Southeast Ethiopia.

From our study, the prevalence rate of HCV was 0.4 %, this is low when compared with 3.1 % in Sudan [15], Tessema et al. 0.7 % [7], Baye et al. 1.7 % [10], Dessie et al. 13.3 % [17], Wasfi and Sadek among Egyptian donors (3.5 %) [6], Yohannes Zenebe et al. 0.6 % [29] among pregnant women, 0.9 % prevalence among population and 1.3 % among adults over 15 years old [30] 0.8 % [25], Anagaw et al. 1 % among medical waste handlers [22], Bekele Sharew et al. 0.61 % [21], and Taye et al. 3.6 % [28].

Our finding is higher than Rishi Diwan and Manu Mathur found no cases of HCV infection [31], Anagaw et al. [22] which was no cases among non medical waste handlers.

The low prevalence of HCV when compared with HBV might be due to the fact that HCV is less infective when compared with HBV and HCV is transmitted mainly through transfusion of blood or blood products, intravenous drug abuse and needle sharing which may not common in Ethiopian Somali Region.

The prevalence of HIV in our study 0.1 % which is lower when compared with national prevalence of 1.9 % for women and 1.0 % for men with an overall prevalence of 1.5 and 1.1 % of Ethiopian Somali Region and study area [32] but higher than study from Egypt in which it was no cases reported [33].

The prevalence of syphilis in our study 0.1 % which is lower than previous studies reported syphilis prevalence ranging from 1 to 10.9 % from Ethiopia in diverse risk groups such as pregnant women, blood donors, street dwellers and elderly people [34].

Even though different study conducted in Ethiopia report co-infection of TTI [7, 18, 22, 25, 30], in this study there was no any co-infection found, the absence of co-infection by HBV, HCV and HIV among blood donors might be due to the presence of small number of HIV, HCV, and syphilis positive donors when compared with HBsAg.

Another approach to improve the safety of blood donation is the introduction of more sensitive test such as the nucleic acid testing (NAT). NAT can detect viraemia and acute viral infections during the serological window period when all serological markers are still negative [14].

NAT is recommended to detect recently infected donors in the window phase of infection, and the main reason for not using in our study area is unaffordable for developing countries like Ethiopia and study area.

In our study area, Malaria screening is not provided routinely due to pre donation interview in which donors having history of malaria cases will be omitted.

There was also statistically significant deference (X^2^ = 8.37, P = 0.01) of HIV virus was seen as the age of donors increased the infection of the virus also increased this may suggest requirement of younger blood donors.

Statistically significant deference in the number of donation and TTI observed from year to year in Jijiga blood bank of Ethiopia Somali Region, attention is needed by decision maker toward knowing the exact situation of the virus in the community.

In our study seroprevalence of TTIs was higher among male donors compared to female donors. The findings may be due to less number of females donating blood; therefore fewer females are screened compared to males.

Even though the trends of TTI as well as hepatitis B fluctuating from year to year, the high prevalence of hepatitis B infections in Ethiopian Somali Region suggests further community based studies to identify the main risk factor exposing Somali communities for blood-borne infections and to develop population-specific interventions to interrupt transmission.

The majority of donors at Jijiga blood banks were replacement (family) donors, a low proportion of voluntary donors may be the indication of a lot of activities are expected from regional health bureau to attain 100 % voluntary donors.

## Conclusions

The present study clearly shows a high seroprevalence of TTIs among blood donors at Jijiga blood bank of Ethiopian Somali Region. Our study showed the increase in HBV prevalence, poor women participation, and a low proportion of voluntary donors in blood donation activities.

### Recommendation

Promoting the culture of voluntary donors, recruitment of female blood donors and proper testing of donor’s blood by using standard methods are recommended.
